# Preparing for Colonoscopy in People with Diabetes: A Review with Suggestions for Clinical Practice

**DOI:** 10.1093/jcag/gwac035

**Published:** 2022-12-30

**Authors:** Alexandra Chirila, Mary E Nguyen, Jill Tinmouth, Ilana J Halperin

**Affiliations:** Department of Medicine, University of Toronto, Toronto, Ontario, Canada; Department of Medicine, University of Toronto, Toronto, Ontario, Canada; Department of Medicine, University of Toronto, Toronto, Ontario, Canada; Sunnybrook Health Sciences Centre, Toronto, Ontario, Canada; Department of Medicine, University of Toronto, Toronto, Ontario, Canada; Sunnybrook Health Sciences Centre, Toronto, Ontario, Canada

**Keywords:** Bowel preparation, Colonoscopy, Diabetes, Dietary modification, Glucose monitoring, Medication management

## Abstract

People with diabetes have an increased risk of adverse events during the peri-colonoscopy period, including hypoglycemia, lactic acidosis, diabetic ketoacidosis and acute kidney injury. This is secondary to inadequate dietary modification, the bowel preparation and antihyperglycemic agent modification. With the availability of many new diabetes agents, endoscopists need updated guidance. This review of current literature provides a practical approach to antihyperglycemic agent modification in the context of colonoscopy preparation, as well as guidelines on dietary changes, the bowel preparation itself and glucose monitoring.

## INTRODUCTION

Colonoscopy is a procedure with both diagnostic and therapeutic utility. It is used to diagnose and treat various abnormalities of the lower gastrointestinal (GI) tract and is integral to colorectal cancer screening, either as a diagnostic procedure following an abnormal fecal occult blood test or as a primary screening test (1, 2). Diabetes is a common condition that is associated with colorectal neoplasia, yet colonoscopy is less effective in people with diabetes (PWD) (3,4). PWD are also at an increased likelihood of complications, including perforation and antihyperglycemic agent (AHA)-associated complications (5). Furthermore, PWD constitute a large proportion of the Canadian population, with a prevalence of 9.7% of adults ages 50 to 64 and 18.1% of adults ages 65 and older having a diagnosis of diabetes in 2020 (6). This suggests that 10% to 20% of patients undergoing colonoscopy have diabetes. Similarly, there has been a surge of new diabetes medications approved in Canada in the past 10 years, including a new drug class, sodium-glucose transporter-2 inhibitors (7). As such, there is a critical need for updated colonoscopy preparation guidelines for PWD, which consider the pharmacologic properties and associated risks of all AHAs currently approved in Canada.

Colonoscopy is more potentially complex for PWD for a variety of reasons, including a higher likelihood of suboptimal bowel preparation and metabolic disturbances (e.g., fasting-mediated water and electrolyte imbalance), and complications associated with inappropriate dose adjustment of AHAs by the endoscopist, prescribing physician or lack thereof (8,9). Without proper dose adjustment to AHAs used to treat both type 1 and type 2 diabetes, PWD can develop hyperglycemia, hypoglycemia, diabetic ketoacidosis, lactic acidosis and/or acute kidney injury (AKI) (9–12).

There is a need to update guidance for the management of PWD undergoing colonoscopy for several reasons. Open-access colonoscopy, when there is no antecedent appointment with the endoscopist before the colonoscopy appointment, has become increasingly common in Canada (13). Without a pre-colonoscopy assessment, preparation and risk assessment may be inadequate. Additionally, old perioperative recommendations to cease all AHAs on the day of a procedure or to hold oral AHAs during the clear fluid diet phase are outdated. Given the risk profile of the new AHA classes that have become available in Canada, these practices put PWD at risk (14,15). Furthermore, both diabetes and obesity (often co-existent with diabetes) are associated with an increased risk of developing colorectal adenomas, and diabetes has shown to be a risk factor for missed adenoma, even on repeat colonoscopy (3,4,16).

Given the elevated risks of colonoscopy, the required preparation for PWD and the need to address the differing dose adjustments for each class of AHAs, the following recommendations have been developed in order to support safer delivery of colonoscopies in patients with both type 1 and type 2 diabetes mellitus. Preparation for colonoscopy in PWD typically involves dietary modification, large bowel lavage, medication management and glucose monitoring, further described as follows. These guidelines have been developed primarily for endoscopists to adequately prepare PWD for colonoscopy, but when endoscopists are not comfortable to make recommendations about specific medication changes, the prescribing physician should be consulted or a referral to a diabetes specialist should be made.

## METHODS

The literature from March 2001 to June 2022 for colonoscopy preparation guidelines for PWD was reviewed using OVID Medline, PubMed and Google Scholar as well as performing a search of the grey literature. Sources included were journal articles, reports, book chapters and webpages of reputable organizations. Key words included colonoscopy, scope, diabetes, diet, dietary modification, bowel preparation, bowel lavage, bowel cleanse, medication, pharmacotherapy and the specific names of each AHA class. Reference sections of the retrieved studies were reviewed for additional resources. In total, 67 relevant articles were identified. These were used to develop the recommendations, which were then reviewed by experts in the field (endocrinology and endoscopy).

## DIETARY MODIFICATIONS IN PWD UNDERGOING COLONOSCOPY

Persons preparing for colonoscopy must follow specific dietary recommendations, which presents specific challenges for PWD. These recommendations in the general population most commonly involve a clear fluid diet starting the day before colonoscopy, or a low-fibre breakfast the day before colonoscopy followed by a clear fluid diet if the procedure is in the afternoon (17). Although not commonly used in practice, a study has shown that a low-residue diet the entire day before colonoscopy was better tolerated than the clear fluid diet without impacting the adequacy of the preparation (18). Although not specific to PWD, this option may facilitate colonoscopy preparation in this population.

If a clear fluid diet is recommended, PWD may choose to consume clear fluids that are low in calories to avoid refined carbohydrates, as is recommended for the management of their diabetes. This hypocaloric diet can cause electrolyte imbalances and even diabetic ketoacidosis (DKA), both of which can cause AKI. Therefore, it should be emphasized to PWD that consuming fluids high in glucose during the clear fluid stage is needed to maintain normal glucose levels. It is recommended that PWD consume 45 g of carbohydrates for meals and 15 to 30 g for snacks, as well as consume fluids with electrolytes. A sample diet can be found in Supplementary Appendix 1.

A normal breakfast the day before colonoscopy before starting clear fluids may be recommended for PWD, as it has shown to improve adequacy of bowel preparation and tolerability in PWD compared to a clear fluid diet starting the whole day before colonoscopy (19). Of note, appropriate AHA adjustments need to be made to prevent hypoglycemia secondary to these dietary modifications.

## LARGE BOWEL LAVAGE IN PWD

High-quality bowel cleansing is essential for the diagnostic accuracy and safety of colonoscopy. Inadequate bowel cleansing can result in cancellation of the procedure and/or need to repeat colonoscopy, increased procedure duration, increased risk of complications and missed lesions, which can develop into colorectal cancers (16,20). The currently recommended bowel preparation for all patients with diabetes mellitus in Ontario is PEG with or without bisacodyl given in a split dose fashion (17). Split-dosing allows the second dose to be taken 4 to 6 hours before the procedure, and has shown a significant decline in the frequency of inadequate bowel preparation in PWD (21). The use of PEG is preferred in PWD as magnesium citrate/sodium picosulphate, citric acid and magnesium oxide preparations are contraindicated in patients with impaired renal function, a common sequela of diabetes (22,23).

A number of studies have shown that diabetes is a predictive factor for inadequate bowel cleansing independent of the protocol used or compliance with preparation instructions (24–28). There are various factors that may contribute to this. First, diabetic neuropathy is associated with poorer bowel preparation, possibly due to the associated constipation, incomplete evacuation, decreased colonic motility and increased colonic transit times (8,29,30). Moreover, PWD has slower gastric emptying, with some AHAs exacerbating this (8,31). Fortunately, diabetes-specific protocols can lead to better bowel preparation. Modifications that can improve bowel preparation include adding lubipristone to PEG, modifying AHA use, delaying the clear fluid diet to 8 hours before colonoscopy and providing patients a specific low-residue diet plan for the days leading up to the procedure (8,19,21,32). It is imperative that adequate hydration with clear fluids is maintained during bowel preparation (i.e., 4 L per day or 250 mL per awake hour) (17).

## DIABETES MEDICATION MANAGEMENT

PWD are at risk of glucose disturbances in the peri-colonoscopy period, and therefore need to adjust their AHAs as well as monitor their blood glucose. A detailed description of the potential issues and peri-colonoscopy recommendations for metformin, glucagon-like peptide 1 receptor agonists, dipeptidyl peptidase IV inhibitors, sodium-glucose cotransporter 2 inhibitors, insulin secretagogues and insulin follows. Some recommendations are based on diet, but for AHAs with a longer half-life, the recommendations are based on the time to the procedure. A summary of the mechanisms of action and associated side effects for all AHAs can be found in Table 1. The recommendations for AHA adjustments are summarized in Table 2. The specific agents in each AHA class can be found in Tables 1 and 2.

**Table 1.** Mechanism of action and adverse effects of antihyperglycemic agents commonly used to treat diabetes

Antihyperglycemic agent		Mechanism of action	Adverse effects
Biguanides		Reduces hepatic gluconeogenesis and increases insulin sensitivity in peripheral tissues.	Risk of lactic acidosis (exacerbated by renal impairment and dehydration), which can develop into acute kidney injury.
Metformin (Glucophage)			
Metformin ER (Glumetza)			
GLP-1 agonists		Mimics endogenous GLP-1 incretin hormone, which increases insulin secretion, decreases glucagon secretion, slows gastric emptying and increases satiety.	Gastrointestinal side effects (including nausea, vomiting, diarrhea, delayed gastric emptying and reduced bowel motility) and risk of acute kidney injury.
Dulaglutide (Trulicity)			
Exenatide (Byetta)			
Exenatide ER (Bydureon)			
Liraglutide (Victoza/Saxenda)			
Lixisenatide (Adylxine)			
Semaglutide oral (Rybelsus)			
Semaglutide injection (Ozempic)			
DPP-4 inhibitors		Inhibits degradation of endogenous GLP-1, stimulating insulin release and supressing glucagon secretion.	Accumulates in patients with renal insufficiency.
Alogliptin (Nesina)			
Linagliptin (Trajenta)			
Sitagliptin (Januvia)			
Saxagliptin (Onglyza)			
SGLT-2 inhibitors		Inhibits reabsorption of glucose in the kidneys, thereby increasing urine glucose excretion.	Risk of ketoacidosis, particularly for patients on insulin that stop or reduce their insulin dose during the clear fluid phase of bowel preparation. Mild diuretic effect, which increases risk of developing dehydration.
Canagliflozin (Invokana)			
Dapagliflozin (Forxiga)			
Empagliflozin (Jardiance)			
Sulfonylureas		Stimulates insulin secretion, ultimately lowering blood glucose.	Risk of hypoglycemia.
Gliclazide (Diamicron)			
Gliclazide MR (Diamicron MR)			
Glimepiride (Amaryl)			
Glyburide (Diabeta)			
Meglitinides			
Nateglinide (Starlix)			
Repaglinide (Gluconorm)			
Insulins	Rapid/Short acting	Stimulates peripheral uptake of glucose into cells from blood, and inhibits production and secretion of glucose by the liver.	Risk of hypoglycemia. Risk of hyperglycemia if dosing is inappropriately reduced or held, which can lead to diabetic ketoacidosis.
	Aspart (Novorapid/Trurapi)		
	Faster insulin aspart (Fiasp)		
	Glulisine (Apidra)		
	Lispro (Admelog/Humalog)		
	Regular human insulin (Humulin R/Novolin ge Toronto)		
	Human biosynthetic insulin (Entuzity)		
	Intermediate acting		
	NPH (Novolin ge NPH)		
	First-generation basal		
	Glargine (Lantus/ Basaglar)		
	Detemir (Levemir)		
	Second-generation Basal		
	Glargine U300 (Toujeo/Toujeo Doublestar)		
	Degludec U100 and U200 (Tresiba)		

DPP-4, Dipeptidyl peptidase IV; ER, Extended release; GLP-1, Glucagon-like peptide 1; MR, modified release; SGLT-2, sodium-glucose Cotransporter 2.

**Table 2.** Antihyperglycemic agent dosing recommendations during colonoscopy preparation and the associated risks

Antihyperglycemic agent	3 days before colonoscopy	2 days before colonoscopy	Day before colonoscopy	Day of colonoscopy	Risks
Biguanides	Continue as normal.	Continue as normal.	Stop taking once clear fluid diet starts.^*^	Resume once meals have resumed.	Risk of lactic acidosis (rare).
Metformin (Glucophage)					
Metformin ER (Glumetza)					
GLP-1 agonists	Continue as normal.	Continue as normal. If taking once weekly injectables, hold starting **two** days before the procedure.^†^	Stop taking once clear fluid diet starts.^*^	Resume once meals have resumed. Once weekly injectable should be taken the evening of the procedure if it was held before the procedure.	Reduced bowel motility.
Dulaglutide (Trulicity)					
Exenatide (Byetta)					
Exenatide ER (Bydureon)					
Liraglutide (Victoza/Saxenda)					
Lixisenatide (Adylxine)					
Semaglutide oral (Rybelsus)					
Semaglutide injection (Ozempic)					
DPP-4 inhibitors	Continue as normal.	Continue as normal.	Continue as normal.	Stop morning of the procedure. Resume the evening after the procedure.	
Alogliptin (Nesina)					
Linagliptin (Trajenta)					
Sitagliptin (Januvia)					
Saxagliptin (Onglyza)					
SGLT-2 inhibitors	Stop taking.^‡^	Already stopped.	Already stopped.	Resume once meals and adequate hydration have resumed.	Risk of ketoacidosis, particularly for patients on insulin that stop or reduce their insulin dose during the clear fluid phase of preparation.
Canagliflozin (Invokana)					
Dapagliflozin (Forxiga)					
Empagliflozin (Jardiance)					
Sulfonylureas	Continue as normal.	Continue as normal.	Stop taking.^‡^	Resume once meals have resumed.	Hypoglycemia.
Gliclazide (Diamicron)					
Gliclazide MR (Diamicron MR)					
Glimepiride (Amaryl)					
Glyburide (Diabeta)					
Meglitinides	Continue as normal.	Continue as normal.	Stop taking once clear fluid diet starts.^*^	Resume once meals have resumed.	Hypoglycemia.
Nateglinide (Starlix)					
Repaglinide (Gluconorm)					
Insulins Rapid/Short acting	Continue as normal	Continue as normal	If taking a fixed dose, take 50% of usual dose once the clear fluid diet starts.* If insulin dosing is based on insulin to carbohydrate ratio, continue typical rapid-acting insulin dosing	Resume once meals have resumed.	Hypoglycemia and possible risk of ketoacidosis (if dose is reduced or held).
Aspart (Novorapid/Trurapi)					
Faster insulin aspart (Fiasp)					
Glulisine (Apidra)					
Lispro (Admelog/Humalog)					
Regular human insulin (Humulin R/Novolin ge Toronto)					
Human biosynthetic insulin (Entuzity)					
Intermediate acting	Continue as normal.	Continue as normal.	Take **80%** of the normal dose.	Take **50%** of the normal dose the **morning** of the procedure. Once eating regular meals, resume normal dose at next scheduled dose.	
NPH (Novolin ge NPH)					
First-generation Basal	Continue as normal.	Continue as normal.	Take **80%** of the normal dose.	Take **50%** of the normal dose the **morning** of the procedure. Once eating regular meals, resume normal dose at next scheduled dose.	
Glargine (Lantus/ Basaglar)					
Detemir (Levemir)					
Second-generation Basal	Continue as normal.	Continue as normal.	T1D patients should take **50–80%** of their normal dose, and T2D patients should take **50%** of their normal dose.	If insulin dose is before the procedure, T1D patients should take **50–80%** of their normal dose, and T2D patients should take **50%** of their normal dose. Once eating regular meals, resume normal dose at next scheduled dose.	
Glargine U300 (Toujeo/Toujeo Doublestar)					
Degludec U100 and U200 (Tresiba)					

DPP-4, Dipeptidyl peptidase IV; ER, Extended release; GLP-1, Glucagon-like peptide 1; MR, Modified release; SGLT-2, Sodium-glucose Cotransporter 2; T1D, type 1 diabetes; T2D, type 2 diabetes.

^*^For antihyperglycemic agents that should be stopped or dose adjusted when the clear fluid diet begins, if a patient eats a normal breakfast the day before colonoscopy (i.e., before clear fluids) and is taking a medication that is dosed more than once per day, the morning dose should be taken before holding the rest of the doses, and if a patient is allowed a low residue diet the day before colonoscopy, medications should be continued as normal until the day of colonoscopy when the clear fluid diet begins.

^†^Only if a dose is scheduled during the 2 days before colonoscopy, if earlier in the week can take the once weekly injectable as scheduled.

^‡^Do not take any doses of AHA during the indicated number of days prior to the scheduled colonoscopy.

### Hypo and Hyperglycemia

Hypoglycemia is a very serious adverse event that can cause significant morbidity, such as confusion, loss of consciousness, seizures and can be fatal (33,34). While most AHAs carry a very low risk, especially when taken as indicated (35–38), insulins and insulin secretagogues carry a notable risk of hypoglycemia. It is critical that PWD are educated on the signs and symptoms of hypoglycemia, but with recurrent episodes of hypoglycemia, patients reach lower glucose levels before experiencing symptoms (33). Therefore, modifying AHAs that elevate insulin is imperative in PWD undergoing colonoscopy, especially since carbohydrate intake is altered during colonoscopy preparation.

In contrast, fear of hypoglycemia causes some patients to stop their AHAs prematurely or take them less often than indicated. Similarly, some providers recommend stopping or decreasing the dose of AHAs prematurely before colonoscopy. These prolonged disruptions of AHAs increase the risk of patients becoming hyperglycemic, which can exacerbate dehydration and cause metabolic decompensation, osmotic diuresis and even AKI (39,40). These risks highlight the importance of glucose monitoring in the peri-procedural period.

### Metformin

Metformin, the first-line pharmacotherapy for type 2 diabetes, is the most commonly prescribed AHA (41,42). More recently, new formulations have been developed, such as extended-release metformin, which reduces side effects and improves adherence (43). Metformin use has been associated with improved cardiovascular outcomes, decreased all-cause mortality and has been shown to exhibit anticancer effects, favouring its use (44,45).

Metformin is renally excreted and can accumulate in the setting of renal impairment, and possibly even lead to lactic acidosis (11). Dehydration also increases the risk of developing lactic acidosis, which can be exacerbated by diuretics, prolonged fasting, colonoscopy preparation, vomiting, diarrhea and excess sweating (41,46). There have been several cases of metformin-associated lactic acidosis (MALA) in PWD following colonoscopy, most of which developed into AKI (11,12,41). This complication is rare but potentially fatal, and is more common in patients taking higher than the recommended dose of metformin (38). Most cases of MALA occurred when metformin was not stopped before bowel preparation, emphasizing the importance of dose adjusting before colonoscopy (12).

*Recommendation*: Metformin should be stopped when the clear fluid diet begins (timing varies depending on what specific dietary recommendations are made for each patient). Once meals have resumed, metformin should be resumed at the normal dose and interval.

### Glucagon-like Peptide 1 Receptor Agonists (GLP-1 RAs)

GLP-1 RAs are used as a second-line therapy for diabetes. Most commonly, the once-weekly injectable GLP-1 RAs are used, including Semaglutide and Dulaglatide. In addition to glucose lowering, these medications result in significant weight loss and a reduction in cardiovascular events (47,48).

The most common side effects of GLP-1 RAs are its effects on the GI tract (e.g., nausea, diarrhea, vomiting and delayed gastric emptying) (49). The increased risk of developing preprocedure nausea with GLP-1 RAs versus insulin leads to poorer patient experience and an increased incidence of rescheduled colonoscopy (50). Another study found that delayed gastric emptying does not appear to have an impact on the adequacy of bowel preparation, but further investigation is required as this study did not examine the effects of different classes of GLP-1RAs despite their differing effects on the GI tract (51). Furthermore, GLP-1 RA use has been linked to the development of AKI, largely due to intravascular depletion secondary to fluid loss with vomiting and diarrhea and decreased fluid intake, which can be exacerbated during colonoscopy bowel preparation (49).

*Recommendation*: GLP-1 RAs should be stopped when the clear fluid diet begins. Once meals have resumed, GLP-1 RAs should be resumed at the normal dose and interval. If a patient takes a once-weekly injectable GLP-1 RA, the dose should be held if it is scheduled within two days before colonoscopy. If held, it should be resumed on the evening of the procedure.

### Dipeptidyl Peptidase IV (DPP-4) Inhibitors

DPP-4 inhibitors are used as second line or adjunct therapy when metformin is not well tolerated or does not provide an adequate therapeutic response, respectively (36). DPP-4 inhibitors are associated with few adverse effects and are weight neutral (36,37,52). DPP-4 inhibitors are renally excreted and therefore accumulate in patients with renal insufficiency (53). Nonetheless, DPP-4 inhibitors are safe to use in patients with impaired renal function as long as appropriate dose adjustments are made (36,53).

*Recommendation*: DPP-4 inhibitors should be stopped the morning of the procedure, and subsequently resumed at the normal dose and interval the evening after the procedure (i.e., same day).

### Sodium-glucose Cotransporter 2 (SGLT-2) Inhibitors

SGLT-2 inhibitors are a newer class of AHAs that are used as second-line therapy (54). They result in weight loss and are beneficial for preventing hospitalization for heart failure, cardiovascular death and progression of nephropathy (55–58).

SGLT-2 inhibitors exhibit a mild diuretic effect, which can increase the risk of dehydration during colonoscopy preparation. Reports have also found that SGLT-2 inhibitors can cause AKI, and that they should be held when a patient is unable to maintain hydration (59). Furthermore, a rare but important risk associated with SGLT-2 inhibitor use is DKA (10). Risk factors of DKA include advanced diabetes, previous DKA, fasting, low-carbohydrate diet, dehydration, insulin insufficiency, reducing or omitting insulin and procedural or surgical stress (10,37,60). Therefore, it is important for patients to be educated on the symptoms of DKA, and monitoring of ketones should be considered during high-risk periods. One study found that the most effective method of avoiding DKA in patients taking SGLT-2 inhibitors is to hold the drug in any circumstance where risk factors that may precipitate DKA are present (60,61). It is necessary to note that SGLT-2 inhibitors can cause euglycemic DKA. Therefore, in patients with signs and symptoms of DKA but normal glucose levels, a bicarbonate and anion gap assessment is critical to not miss the diagnosis (54).

*Recommendation*: SGLT-2 inhibitors should be stopped starting 3 days before colonoscopy (i.e., no doses should be taken during the full 3 days before), regardless of when the clear fluid diet begins. Given the long half-life of SGLT-2 inhibitors, their effects can persist for many days (60). Once meals and adequate hydration have resumed, SGLT-2 inhibitors should be resumed at the normal dose and interval.

### Sulfonylureas and Meglitinides (Insulin Secretagogues)

Insulin secretagogues, which include sulfonylureas and meglitinides, are second-line therapies for diabetes. They are low in cost and elicit a rapid glucose-lowering response (58). As this class of AHAs stimulate insulin release regardless of insulin levels, they come with a risk of hypoglycemia (62). It is important to recognize that some sulfonylureas have a long half-life and can cause hypoglycemia even if taken more than 24 hours before colonoscopy (9).

*Recommendation*: Meglitinides should be stopped when the clear fluid diet begins. Sulfonylureas should be stopped starting the full day before colonoscopy. Once meals have resumed, sulfonylureas and meglitinides should be resumed at the normal dose and interval.

### Insulins

Insulin is the mainstay of treatment for type 1 diabetes and is also used in patients with type 2 diabetes who have failed on non-insulin AHAs (39). Insulin therapy reduces glucose regardless of blood glucose levels, resulting in a risk of hypoglycemia if not dosed properly (63). A careful balance is required when dose-adjusting insulin for PWD, as it has a narrow therapeutic index (64). As insulin regimens reflect typical carbohydrate intake, dietary modification during colonoscopy preparation requires insulin dose adjustment to avoid hypoglycemia. In contrast, inappropriate insulin omission or dose reduction, particularly in combination with a hypocaloric diet followed by fasting and laxative use, can lead to the development of DKA (9,65). To minimize these adverse events, it is recommended to maintain glucose between 3.9 and 10.0 mmol/L at least 70% of the time (66).

*Recommendation*: Insulin dosing must be reduced or omitted before colonoscopy. For rapid- and short-acting insulins, patients should decrease their normal doses by 50% once the clear fluid diet begins. If a patient doses their insulin based on insulin to carbohydrate ratio, they should continue with their typical rapid-acting insulin dosing. For intermediate-acting and first-generation basal insulins, patients should take 80% of their normal dose the full day before the procedure, and 50% of their normal dose the morning of the procedure. For second-generation basal insulins, people with type 1 diabetes should take 50-80% of their normal dose, and people with type 2 diabetes should take 50% of their normal dose, both starting the day before the colonoscopy. Premixed insulin doses should be reduced by 50% at breakfast and dinner the day before colonoscopy. Once meals and adequate hydration have resumed, all insulins should be resumed at the normal dose and interval. For patients with type 1 diabetes, basal insulin should never be omitted, as this poses a significant risk for DKA.

For patients wearing an insulin pump, the basal insulin rate should be reduced to 80% of their normal rate once the clear fluid diet begins. Once meals have resumed, insulin pump dosing should be resumed at the normal dose and interval. For patients using automated insulin delivery, selecting the exercise setting may provide added protection from lows during the preparation period. There is conflicting guidance on the safety of keeping an insulin pump on during a procedure involving cautery. It is recommended that patients consult the user guide or their pump manufacturer directly if there is a chance cautery could be used during colonoscopy. The insulin pump should not be removed for longer than one hour as this poses a significant risk of developing DKA.

## GLUCOSE MONITORING

Patients should monitor their glucose before all meals and at bedtime the day before, and every 4 hours starting at 07:00 am the day of the procedure, as well as anytime symptoms of hypo- or hyperglycemia are experienced. PWD should be advised that if their glucose drops below 5 mmol/L, they should consume high-glucose fluids, and if their glucose rises above 10 mmol/L, only glucose-free fluids should be consumed (15). PWD should also bring their glucose meter, test strips, and low glucose treatment (e.g., dextrose tablets, apple juice drink boxes) with them on the day of the procedure. A point of care capillary glucose should be done before the initiation of sedation; if the glucose values are in a safe range (i.e., 5 to 12 mmol/L) then it is safe to assume they will remain so for the duration of the procedure. If the levels are outside of that range than more frequent monitoring by a nurse-obtained capillary sample may be necessary. PWD should be encouraged to monitor their glucose more frequently in the few days following the procedure.

If a PWD wears a continuous glucose sensor they will be able to view glucose readings more frequently during the procedure provided they have their smart phone, reader or receiver with them. The sensor should not be removed at any point during the peri-procedural period as glucose may fluctuate due to changes in diet and AHA dosing. During the procedure, glucose can be monitored by a member of the healthcare team. If light sedation is used, which is increasingly recommended as it is associated with a lower incidence of cardiorespiratory complications and faster recovery, the patient can monitor their sensor glucose reading themselves during the procedure (67).

## TIMING OF PROCEDURE

There is evidence that the timing of colonoscopy may impact the adequacy of bowel preparation. Typically, early morning procedures have been favoured, as this allows for a decreased fasting period and earlier resumption of AHAs, believed to minimize the risk of adverse events such as hypo- or hyperglycemia, but there is no evidence to support this (9). Conversely, mid-morning procedures allow for more convenient split dosing of the laxative regimen, as the second dose should be taken 4 to 6 hours before colonoscopy (21). Similarly, afternoon procedures where the patient is allowed to consume a low-fibre breakfast the morning of the procedure revealed a significant improvement in the rate of adequate bowel preparation (19).

## CONCLUSION

Given the rapidly evolving field of diabetes management, updated, evidence-based recommendations regarding colonoscopy for PWD are imperative for minimizing the risk of complications and to maximize procedure efficacy. PWD should be given a personalized diet plan by their healthcare provider for the days leading up to their colonoscopy. Additionally, diabetes-specific protocols for large bowel lavage should be used to decrease the likelihood of inadequate bowel preparation and its associated risks. AHAs must be appropriately adjusted due to the risk of AHA-related complications in the context of colonoscopy preparation. Lastly, PWD should be counselled on monitoring for symptoms of hypo- and hyperglycemia and should frequently check their blood glucose in the peri-colonoscopy period. Supplementary Appendix 1 provides a sample template that primary care providers, gastroenterologists or diabetes care team members can fill out and provide to patients regarding diabetes management during colonoscopy preparation.

These guidelines allow for the use of colonoscopies, which are essential for the diagnosis and management of many conditions and for colorectal cancer screening, in a safe and patient-centered way, addressing the risks unique to PWD.

## Supplementary Material

gwac035_suppl_Supplementary_Appendix

## Data Availability

There are no data associated with this manuscript.
