# Supplementary material for: Preparing for Colonoscopy in People with Diabetes: A Review with Suggestions for Clinical Practice
Source: J Can Assoc Gastroenterol. 2022 Dec 30;6(1):26–36. doi: 10.1093/jcag/gwac035 (PMC9915054; doi:10.1093/jcag/gwac035)
Supplement: gwac035_suppl_Supplementary_Appendix [file gwac035_suppl_supplementary_appendix.docx]

***Appendix 1: Instructions for Colonoscopy Preparation for People with Diabetes***


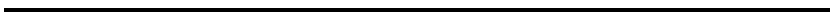


**For people with diabetes, any procedure, such as colonoscopy, that causes you to miss a meal or change your usual meal plan will require special planning to safely manage blood glucose. Some recommendations are as follows:**

- Check your blood glucose level before all meals and at bedtime the day before the procedure and every 4 hours starting at 07:00 on the day of the procedure.
- Check your blood glucose if at any time you have symptoms of low blood glucose (ex. dizziness, sweating, trembling, hunger, fatigue or fast heartbeat) or very high blood glucose (ex. increased thirst, frequent urination, fatigue or fruity smelling breath).
- Bring your blood glucose meter, test strips and a low blood glucose treatment (ex. dextrose tablets, apple juice drink boxes and/or cans of regular ginger ale or Sprite) with you on the day of the procedure.
- You may continue to wear your glucose sensor during the procedure but if directed, you will need to remove it.
- Insulin pumps may need to be removed if there is electrocautery required during your procedure, as it could become damaged. We recommend you check your pump user guide or contact the manufacturer to be sure. Try to minimize the time without your pump to less than one hour (take off your pump just before the procedure begins and reconnect it as soon as possible in the recovery area).

**Clear liquid food choices for the day before your colonoscopy:**

**You are allowed to have a normal breakfast the day before your colonoscopy.**

**Aim for 45 grams of carbohydrate at meals and 15-30 grams of carbohydrate for snacks.**

**Avoid foods that are red or have red dyes (ex. cherry, berry or grape flavors).**

**Food items Grams of carbohydrate**

Apple juice (1/2 cup) 15

White grape juice (1/2 cup) 20

Sports drink such as Gatorade (1 cup) 14

Gelatin (Jello), regular sweetened (1/2 cup) 15

Orange popsicles or ice pops (read the label) 15

Italian ice--not sherbet (read the label) 30

Sugar (for coffee or tea) (1 teaspoon or packet) 4

**Food items without carbohydrate**

Fat-free broth, bouillon, or consommé

Diet clear soda

Coffee

Tea, unsweetened or diet

Seltzer

Flavored water

| **Sample Menu**  Aim for 45 grams of carbohydrate at meals and 15-30 grams of carbohydrate for snacks. | | | |
| --- | --- | --- | --- |
| **Breakfast** | **Lunch** | **Dinner** | **Snacks** |
| Normal breakfast on day before.  Clear fluids on day of colonoscopy, example:  • Apple or white grape juice (1 cup)  • Regular sweetened gelatin (1 cup)  • Tea with lemon | • Consommé (3/4 cup)  • Italian ice (1/2 cup)  • Regular sweetened gelatin (1/2 cup)  • Tea with lemon | • Consommé (3/4 cup)  • Strained fruit juice (1 cup)  • Regular sweetened gelatin (1/2 cup)  • Tea with lemon | ONE OF:  • Apple or white grape juice (1/2 cup)  • Regular sweetened gelatin (1/2 cup)  • Orange popsicle or ice pop  • Italian ice (1/2 cup) |

****This section should be left blank and written in by your healthcare provider****

**Changes to Medication Prior to Colonoscopy**

**☐** **No medication changes required**

**☐ The following medication changes should be performed prior to your colonoscopy**

| **3 days before** |  |
| --- | --- |
| **2 days before** |  |
| **1 day before** |  |
| **Day of procedure** |  |

**Other notes:**
